# A Survey for Human Tissue-Level Determinants of CAV1 Regulation and Function

**DOI:** 10.3390/ijms26083789

**Published:** 2025-04-17

**Authors:** Víctor Jiménez-Jiménez, Fátima Sánchez-Cabo, Martin A. Schwartz, Miguel Sánchez-Álvarez, Miguel Ángel del Pozo

**Affiliations:** 1 Mechanoadaptation & Caveolae Biology Lab, Cell and Developmental Biology Area, Centro Nacional de Investigaciones Cardiovasculares Carlos III (CNIC), 28029 Madrid, Spain; victor.jimenez@cunef.edu; 2 Bioinformatics Unit, Centro Nacional de Investigaciones Cardiovasculares Carlos III (CNIC), 28029 Madrid, Spain; fscabo@cnic.es; 3 Department of Quantitative Methods, CUNEF Universidad, 28040 Madrid, Spain; 4 Yale Cardiovascular Research Center, Section of Cardiovascular Medicine, Department of Internal Medicine, School of Medicine, Yale University, New Haven, CT 06510, USA; martin.schwartz@yale.edu; 5 Department of Cell Biology, Yale University, New Haven, CT 06510, USA; 6 Department of Biomedical Engineering, Yale University, New Haven, CT 06510, USA; 7 Cell Compartmentalization, Homeostasis and Inflammation Lab, Department of Metabolic and Immunity Diseases, Instituto de Investigaciones Biomédicas Sols-Morreale, 28029 Madrid, Spain

**Keywords:** CAV1, transcriptional regulation, immune infiltration, immune crosstalk, SMARCA2, PRC2, TEAD4, RELA, STAT3, MYC

## Abstract

CAV1 is a protein-coding gene linked to several disorders, including cancer, lipodystrophy, and cardiovascular diseases. While its ability to respond to various mechanical and metabolic stimuli has been documented, a comprehensive understanding of its physiological regulation in humans is lacking. We leveraged the comprehensiveness of human post-mortem tissue data from the Genotype-Tissue Expression (GTEx) consortium, systematically exploring the sources of variability in CAV1 transcriptional levels using extensive bulk and single-nuclei RNA-seq datasets. This human-centric approach, avoiding inter-species comparisons, constitutes a unique resource to explore CAV1 regulation within the complexity of human tissues. Notably, cell type proportion was identified as a major determinant of CAV1 transcription levels across tissues. Donor physiological conditions, including disease states and end-of-life circumstances, also exhibited a tissue-specific influence. Among primary upstream regulators associated with CAV1, chromatin modifiers stood out, especially SMARCA2, which showed a positive correlation across tissues, and PRC2 complexes, which exhibited tissue-specific correlation. Upstream regulatory networks determining CAV1 levels are also enriched for annotations such as mechanobiology (e.g., TEAD4), immunity (e.g., RELA and STAT3), and metabolism (e.g., MYC and NRF1). A remarkable observation was a strong correlation between CAV1 and the relative infiltration of immune cells across tissues, supporting a potential role for CAV1 as a marker and driver of tissue immune infiltration.

## 1. Introduction

Caveolae are flask-shaped nanoscale invaginations of the plasma membrane (PM) [1]. Discovered in 1953 by George Palade [2], they exhibit a specific lipid and protein composition, rich in cholesterol and sphingolipids, which confers distinct physicochemical properties. Caveolae have emerged as critical players in regulating membrane tension, mechanotransduction, and metabolism, and are involved in several aspects of cell physiology [3].

Caveolin-1 (CAV1), first discovered in 1989 as a target of protein kinase Src [4,5], was identified in 1992 as a key component for the formation and function of caveolae [6,7,8]. It is essential in non-striated muscle cells for caveolae formation [9,10,11], and its artificial expression can induce the formation of PM invaginations in cells typically lacking caveolae such as lymphocytes [12]. The caveolin protein family, including CAV1, CAV2, and CAV3, exhibits variable conservation across metazoans, whereby some phyla, such as Arthropoda, have lost this gene class; in contrast, caveolae formation and expression of this gene family is a distinctive feature in vertebrates. The cell type specificity of CAV1 and CAV3 isoforms is a fascinating aspect of their biological function. CAV1 and CAV3, while structurally similar, exhibit distinct patterns of expression across different cell types, stressing their specialized roles in cellular physiology. CAV1 expression is detected in many cell types but is particularly prominent in endothelial cells, type I pneumocytes, adipocytes, and activated fibroblasts [13]. Its role in these cells is multifaceted, contributing to caveolae formation, signal transduction, and lipid regulation. In contrast, CAV3 is specifically expressed in cardiac and skeletal myocytes, where it is necessary for caveolae formation [14,15]. Members of the cavin protein family, particularly CAVIN1, identified in 2008, are also crucial for caveolae formation in any cell type [16,17]. These proteins form complexes with caveolins, stabilizing the caveolar structure. However, the roles of CAV1 in cellular physiology and in complex diseases like cancer, cardiovascular diseases, and metabolic disorders extend beyond the structural scaffolding of caveolae, and are particularly notable for its context-dependent nature. In cancer biology, CAV1 can function as both a tumor suppressor and a promoter depending on cancer type and stage [18]. Within caveolae, CAV1 acts as both a membrane-trafficking protein and a scaffolding protein, orchestrating the assembly of signaling molecules. Outside caveolae and independent of CAVIN1, CAV1 broadly influences cellular signaling, with effects on a multiplicity of pathways [19]. CAV1 expression levels also determine the trafficking and distribution of cholesterol and sphingolipids across multiple cell compartments, determining the functional state of the endoplasmic reticulum, mitochondria, or endosomal compartments.

This complexity is further accentuated by the responsiveness of CAV1 transcriptional levels in response to numerous external stimuli. CAV1 regulation and its role as an upstream or downstream regulator are highly specific to a given context. For example, the lack of CAV1 has been linked to increased Sp1 transcriptional and nuclear translocation levels in kidney glomerular mesangial cells [20], while its increase is associated with p53 activation and apoptosis in HeLa cells treated with DHA [21]. CAV1 positively regulates YAP activation in fibroblasts [22], influences SMAD3 phosphorylation and nuclear translocation in endothelial cells [23], and affects NF-κB activity in breast cancer cell lines [24]. Conversely, various upstream regulators of CAV1, such as the GR receptor in mouse myotubes and skeletal muscle [25] and the OXPHOS/p38/Sp1 pathway in fibroblasts [26], have been identified. These studies highlight the cell type- and context-dependent nature of CAV1 regulation.

We aimed to better understand this complexity through a holistic approach using post-mortem expression data exclusively from human tissues provided by the Genotype-Tissue Expression (GTEx) project [27]. Focusing on human tissue-derived data, rather than cross-species comparisons, enables us to model CAV1 regulation and its associated functions across diverse human tissues and pathological conditions, offering a significant advancement in capturing the physiological relevance of CAV1 to humans. Our findings constitute a source of data-driven hypothesis for potential regulatory patterns and candidate upstream factors at the tissue level (thus, including this level of complexity from human samples) that will guide future experimental validation. Our integrative computational approach served as a complementary tool to leverage the richness of the GTEx dataset, helping to map out the landscape of CAV1 transcriptional regulation in a physiologically relevant observational setting. By analyzing this rich dataset, we hope to gain a deeper understanding of the tissue-specific regulation of CAV1, exploring how different regulatory programs might govern its diverse roles in various tissue environments relevant to human disease. A key aspect of our research involved analyzing how CAV1 expression levels correlate with parameters such as health status, age, body mass index (BMI), and other phenotypic characteristics of the GTEx project’s donors. We explored the principles governing CAV1 natural variability across tissues and its upstream regulating networks. Further, we generated models by which CAV1 tissue expression could be partially deconvoluted on cell type relative composition; these studies position CAV1 as an overlooked biomarker and candidate regulator of immune infiltration.

## 2. Results

### 2.1. Enhanced Expression of CAV1 and CAVIN1 in Caveolae-Rich Versus Caveolae-Deficient Tissues

We first investigated whether human tissue transcript levels of CAV1 and CAVIN1, key caveolar components, could distinguish tissues with expected abundant versus scarce caveolae. Using bulk RNA-Seq data from human donors in the GTEx project, we measured the average expression of these two principal non-striated muscle cell caveolae markers across various human tissue types. This exclusive reliance on human data highlights a key strength of our study, providing insights directly applicable to human physiology without potential species-specific effects.

**Figure 1:**
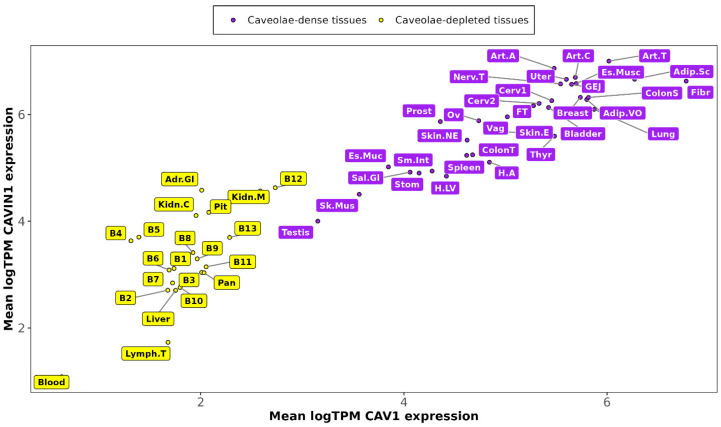
CAV1 and CAVIN1 mean transcriptional levels across GTEx tissue categories. In yellow, tissues expressing low CAV1 expression levels and in purple tissues enriched in CAV1 expression.

As expected, the average transcript levels of CAV1 distinguished tissues traditionally considered caveolae-deficient—such as brain, blood, liver, pancreas, kidney, and adrenal gland, in yellow in Figure 1—and those with a high proportion of caveolae-rich cell types, such as endothelium, smooth muscle, or adipocytes, shown in purple in Figure 1.

It is worth noting that excluding blood and transformed lymphocytes, all the tissues, even those presumed to be caveolae-deficient, contain cell types where caveolae have been previously characterized and express CAV1 and CAVIN1 (Figure 1). This widespread distribution can be attributed to the ubiquitous presence of endothelial cells, a cell type known to be enriched in CAV1.

### 2.2. Understanding the Sources of Variation in CAV1 Expression Across Tissues

Our study identified significant variation in CAV1 expression across tissue samples stemming from complex environmental factors. While we cannot ultimately prove causality, we employed a series of increasingly complex linear models to infer potential causality. By progressively incorporating cell type proportions and additional phenotypic variables, we aimed to distinguish true biological associations from artifacts driven by technical or compositional factors. The specifics of these models are detailed in the Materials and Methods Section.

#### 2.2.1. Influence of Public GTEx Variables on CAV1 Expression

We initially assessed the impact of publicly available covariates from GTEx on CAV1 expression. These covariates encompass RNA quality metrics (including RNA integrity number (RIN), autolysis score, ischemic time, and time in PAXgene™ fixative) and phenotypic variables like gender, age, and terminal phase context. The latter, a scale classifying end-of-life circumstances, offers biological insights into the impact of death causes and conditions on tissue gene expression.

Tissue-specific correlations between each feature and CAV1 expression were identified using multiple linear regression models with a significance threshold of *p* < 0.05. Table 1 reports the number of tissues in which each public GTEx variable reached significance under different modeling scenarios, illustrating how variable significance changes as we incorporate cell type proportions and other phenotypic factors. Notably, terminal phase context significantly correlated (*p* < 0.05) with CAV1 expression in 17 out of 49 tissues when only public GTEx variables are included (model set 1), suggesting effects of the individual’s manner of death. RIN showed significant associations in 15 tissues (*p* < 0.05), suggesting that it may reflect differential RNA degradation linked to cell type variation within tissues.

#### 2.2.2. Cell Type Proportions Predict CAV1 Levels

Before including cell type proportions in our models, the observed correlation between RIN and CAV1 could have been misinterpreted as evidence of a direct biological relationship. However, we considered this initial association to be an artifact driven by technical or compositional confounders rather than a true mechanistic link. To test this assumption, we introduced cell type proportions as predictor variables in the subsequent models. By incorporating these estimates derived from publicly available single-nuclei data from the GTEx project (see Section 4), we found that the influence of RIN on CAV1 levels diminished. This outcome supported our interpretation that the initial RIN-CAV1 correlation primarily reflected underlying differences in tissue composition rather than a causal connection.

In addition to resolving this issue, our analysis of single-nuclei data revealed that CAV1 can serve as a cell type-specific marker. CAV1 was predominantly expressed in type I pneumocytes, adipocytes, pericytes, endothelial cells, and fibroblasts, while it was absent in skeletal muscle myocytes and numerous immune cell subsets. This finding further underscores the importance of considering cellular heterogeneity when interpreting CAV1 expression patterns across diverse tissues.

Thus, cell type proportion is a variable that significantly influenced the predictive power of our models for CAV1 expression, with cell types emerging as primary predictors across all the tissues. This finding highlights the critical role of cell type heterogeneity in determining CAV1 levels. A notable outcome of this revised modeling approach was the diminished significance of the RNA integrity number (RIN) in tissues where it was previously considered a significant factor (see Table 1). Instead, the proportion of specific cell types emerged as a more relevant predictor. This shift suggests that the earlier observed correlations of RIN with CAV1 expression levels in certain tissues were, in fact, reflecting underlying differences in cell type composition. This result supports our hypothesis that RIN can act as a proxy for differential RNA degradation linked to cell type variation in many tissues, validating the concept that the relevance of RIN in previous models was largely attributable to its association with cell type proportions rather than a direct effect on CAV1 transcript levels. Although this modeling approach cannot ultimately prove causality, the stepwise inclusion of cell type proportions and additional covariates allowed us to infer that the initial RIN-CAV1 correlation was driven by confounding factors rather than a direct regulatory mechanism. This insight underscores the importance of considering cellular heterogeneity in genomic studies, as it can significantly influence gene expression profiles and potentially lead to more accurate interpretations of molecular data.

#### 2.2.3. Tissue-Specific CAV1 Expression and Physiological Conditions

Our study delved into the GTEx dataset to explore multiple variables on CAV1 expression, employing a rigorous analytical approach that included elastic net feature selection to handle the complex and collinear nature of the data. This methodology, which also considered cell type proportions as a crucial factor, yielded findings that warrant further discussion.

The analysis confirmed some well-known determinants of CAV1 levels while revealing new associations and clarifying previously contradictory findings. For instance, the study highlighted the responsiveness of CAV1 expression, particularly in response to environmental factors, in the skin, as evidenced by the significant influence of race and skin type. A white racial background was associated with higher levels of CAV1 in both sun-exposed and non-sun-exposed skin. Additionally, variations in CAV1 expression were linked to melanocyte proportion and seasonal changes, indicating CAV1′s involvement in skin melanin regulation [28].

In terms of body composition, we observed that BMI was a strong predictor of CAV1 levels in adipose tissues. This finding agrees with previous reports on a direct but minor impact of BMI on CAV1 expression [29,30].

The study also uncovered new associations of CAV1 with disease states. For instance, lower CAV1 levels in lung tissue were related to chronic obstructive pulmonary disease (COPD), while an abnormal white blood cell count was associated with higher CAV1 levels in various brain regions. Additionally, gender emerged as a predictor of CAV1 levels in thyroid tissue, which could reflect gender-specific physiological variations in thyroid tissue composition.

One of the most striking findings was the significant role of the terminal phase context in predicting CAV1 levels across multiple tissues. This variable or other death-related variables, such as Death Manner, Death Place, Cohort (organ donor, post-mortem donor, or surgical donor), or Death Connected to a Ventilator, indicative of the end-of-life circumstances, remained a consistent predictor across all the models even after adjusting for cell type composition (see Table 1). The findings also indicated that variables like RIN, previously thought to be significant, lost their predictive power in many tissues when cell type proportions were considered, supporting our hypothesis that RIN is a proxy for cell type differential degradation (see Table 1 below).

Our analyses indicate that terminal phase context, a key variable in the GTEx dataset, significantly influences CAV1 expression levels across various tissues. This influence is not merely a technical artifact but seems to reflect biologically relevant aspects of tissue physiology beyond the effects of sample cellular composition. For example, in muscle tissues, the lowered CAV1 levels in individuals dying on a ventilator could be attributed to the absence of mechanical stimulation in the treatment period before death, highlighting the potential role of CAV1 in the response to mechanical changes by these tissues. Similarly, in visceral and subcutaneous adipose tissue, the lower CAV1 levels in ventilated donors might be linked to alterations in metabolic activity at the time of death. In contrast, digestive tract tissues like the gastroesophageal junction and esophagus muscularis show higher CAV1 expression in ventilated individuals, possibly due to reduced intestinal activity in cases of intravenous feeding. These observations underscore the complex and context-specific role of CAV1 in tissue physiology, beyond the technical aspects of sample collection and handling.

**Figure 2:**
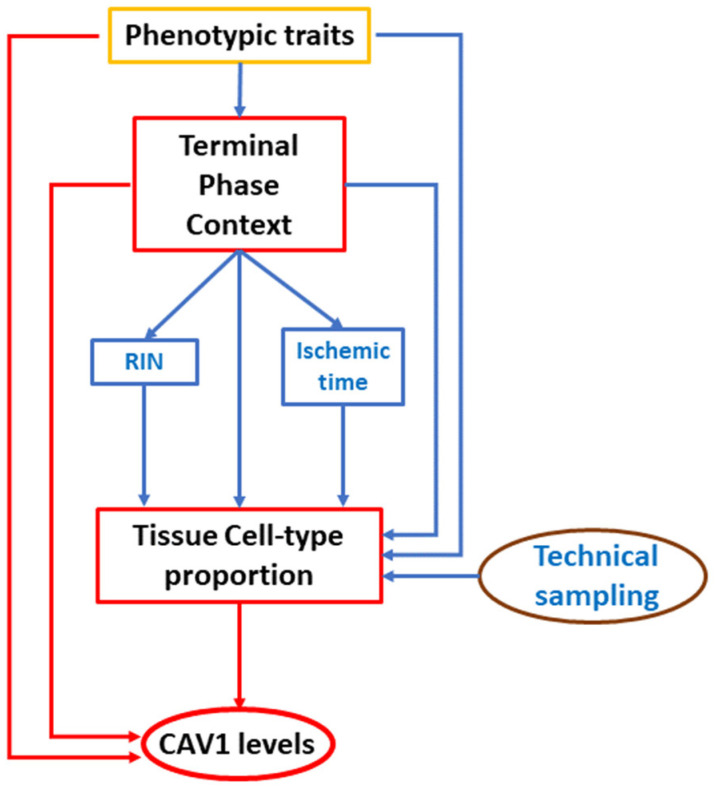
DAG model for GTEx variable relationships with CAV1 levels.

Our model, shown in the directed acyclic graph (DAG) in Figure 2, suggests a complex interplay of health-related phenotypic variables and technical factors in determining CAV1 levels. The terminal phase context, influenced by various phenotypic traits, significantly impacts RNA integrity number (RIN) and ischemic time, which are associated with variations in cell type proportion. These changes in cell type proportion are directly linked to CAV1 expression levels. We also observed that RIN and ischemic times, particularly for individuals who died while on a ventilator, differ significantly from those who died suddenly. This difference likely results from the reduced time between death and sample collection for individuals who died connected to a ventilator inside the hospitals. The model posits that these factors collectively modify CAV1 levels by influencing the composition and activity of different cell types within tissues. As a result, understanding the source of variation in CAV1 expression is crucial since it arises from regulatory processes that respond to these sources of variability, thereby altering CAV1 levels and those of co-regulated transcripts. By incorporating cell type proportions and additional phenotypic data in a stepwise modeling framework, we moved closer to a form of causal inference commonly employed in observational studies. While this approach cannot fully prove causality, it is one of the most robust statistical strategies available to identify potential causal relationships when controlled experiments are not feasible. Modeling how these interconnected factors can shape CAV1 expression helps to clarify that variation arises from regulatory processes responsive to these sources of variability, ultimately influencing CAV1 levels and the expression of co-regulated genes. In the following section, we delve deeper into co-expression networks to identify candidate upstream regulators that may mediate these observed relationships.

### 2.3. Global Analysis of Tissue-Specific CAV1 Correlation Vectors (TSCVs) Fails to Differentiate Caveolae-Rich from Caveolae-Deficient Tissues

**Figure 3:**
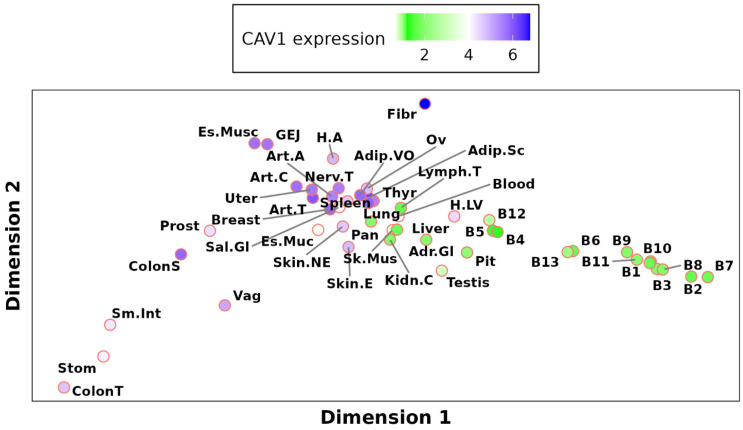
Multidimensional Scaling (MDS) of CAV1 Tissue-Specific Correlation Vectors (TSCVs). MDS was performed as described in Materials and Methods Section 4.4.1, resulting in Dimension 1 and Dimension 2, which represent the principal axes of variation summarizing the high-dimensional correlation data. These dimensions do not correspond to individual biological parameters but rather highlight patterns of similarity and difference among tissues. The color scheme represents the mean CAV1 expression in the samples associated with each tissue corresponding to a specific CAV1 TSCV.

Our study analyzed the complex tissue-specific regulatory patterns of CAV1 by examining CAV1 Tissue-Specific Correlation Vectors (TSCVs). A TSCV represents the co-expression pattern of genes with CAV1 in a specific tissue. We hypothesized that tissues rich in caveolae might show distinct CAV1 regulatory patterns compared to those with fewer caveolae. To explore this, we employed Multidimensional Scaling (MDS) analysis to summarize the correlation patterns, obtaining Figure 3. For a detailed explanation of the MDS procedure, how dimensions were derived, and how to interpret them, please see Multidimensional Scaling Analysis (MDS) section.

In our MDS analysis, we observed an apparent clustering of brain tissues on one side of the plot. Initially, this seemed to indicate a distinct regulatory pattern. However, upon reducing the overrepresentation of brain samples by including only three representative brain tissues, this clustering vanished (see Supplementary Figure S1). This modification integrated brain tissues with the rest of the tissues in the analysis, showing a more homogeneous distribution across the MDS plot. This outcome indicates that the initial separation of brain tissues was not reflective of unique CAV1 regulatory programs, but rather a result of their disproportionate representation in the dataset.

**Figure 4:**
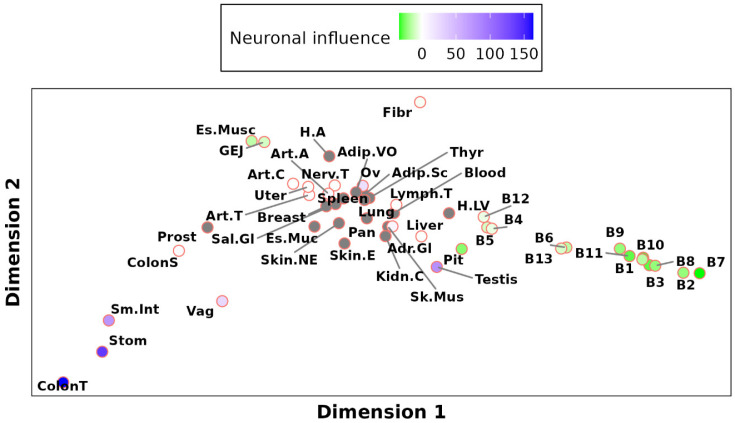
Neuronal Importance Projection on MDS Plot. This plot illustrates the correlation between the CAV1 levels and neuronal cell type proportions across tissues. Tissues where the CAV1 levels positively correlate with the neuronal content are shown in blue, while those with an inverse correlation are in green. Tissues where the neuronal content does not significantly predict CAV1 expression are colored white. Tissues lacking neurons are marked in gray, indicating their non-contribution to the prediction of CAV1 levels.

Notably, the analysis revealed a significant correlation between the variability in TSCVs and the ability of neuronal (Figure 4) and lymphocyte (Supplementary Figure S2) content in the sample to predict CAV1 levels. In tissues on one side of the MDS plot, neuronal content positively predicted CAV1 levels, while on the other side, an inverse relationship was observed. Lymphocyte content showed an opposite pattern (Supplementary Figure S2).

This intriguing finding implies that the differences among TSCVs are not driven by the overall caveolae density but potentially by other factors like the neuronal and lymphocyte content. It suggests a more complex scenario where CAV1 regulation is influenced by cell type composition, challenging our initial hypothesis of distinct regulatory networks based solely on caveolae presence. External stimuli altering cell type proportions in tissues may have a more pronounced impact on our findings than stimuli specifically targeting CAV1 levels. However, this complexity does not negate all results, as the highest correlations with CAV1 across all the TSCVs consistently involve caveolae members such as CAV2, CAVIN1, and EHD2. This consistency underscores the relevance of caveolae components in CAV1 regulation but also emphasizes the need for the careful consideration of cell type composition when interpreting the functions and upstream regulators significantly associated with each CAV1 TSCV.

#### 2.3.1. CAV1 Tissue-Specific vs. Ubiquitous Functions

We performed a gene set enrichment analysis of CAV1 TSCVs to explore their varied roles across tissues. Despite CAV1’s involvement in diverse biological processes, cellular components, and molecular functions, its functional contribution varies significantly among tissues. Notably, a substantial portion of gene ontology biological processes (81.7%), cellular components (87.9%), and molecular functions (66.9%) were found to be CAV1-enriched in at least one tissue.

**Figure 5:**
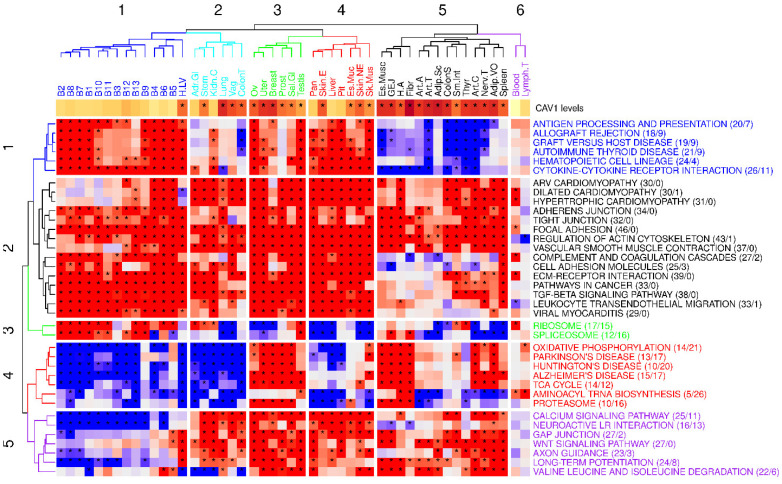
Heatmap of the KEGG pathways significantly enriched in 25 or more tissues from GTEX. Asterisks denote the KEGG pathways that are found to be significant, with an adjusted B-H *p*-value of 0.05. For each pathway, the number of tissues in which it is significantly enriched in genes positively correlated with CAV1, and the number of tissues in which it is significantly correlated with genes inversely correlated with CAV1, are provided in parentheses. The heatmap color coding represents the direction of enrichment in tissue-specific gene correlation vectors: red indicates a positive enrichment, meaning the pathway is associated with genes positively correlated with CAV1 expression in that tissue, whereas blue indicates a negative enrichment, meaning the pathway is associated with genes inversely correlated with CAV1 expression. Lighter shades reflect weaker enrichment, while darker shades represent stronger enrichment. The topmost upper row, depicted in shades of brown, represents the mean CAV1 level; the darker shades of brown indicate higher CAV1 levels. An asterisk designates whether that tissue expresses high or low CAV1 levels, in accordance with the clustering shown in Figure 1.

We identified clusters of pathways correlating with CAV1 across tissues (see Figure 5 summarizing the results). One key cluster was associated with mechanosensing and adaptation, present ubiquitously across tissues. Other clusters, however, showed tissue-specific correlations with CAV1, particularly in immune response and mitochondrial function. Contrary to our initial hypothesis, our analysis did not find any clear tissue clustering based on caveolae presence, indicating a more intricate regulatory landscape for CAV1.

Remarkably, the “Focal adhesion” pathway, linked with mechanosensing, positively correlated with CAV1 expression in most tissues, highlighting the pivotal role of CAV1 in cell–substrate interaction processes. The analysis also recapitulated varying tissue- and context-dependent associations with mitochondrial and immune functions.

This comprehensive functional analysis of CAV1 underscores its broad influence across different cellular contexts. It reveals a significant role for CAV1 in mechanical and mechanoadaptive processes, and its context-dependent involvement in mitochondrial and immune functions, shaped by diverse regulatory networks and stimuli.

#### 2.3.2. Inferring Transcriptional Regulators in CAV1-TSCVs

**Figure 6:**
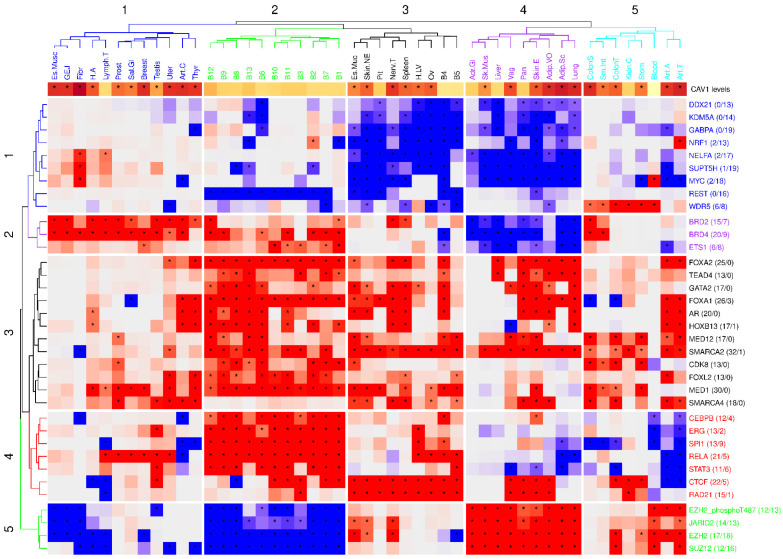
Heatmap of transcriptional regulators significantly enriched in 13 or more tissues from GTEX. Asterisks denote the transcriptional regulators that are found to be significant, with an adjusted B-H *p*-value of 0.05. For each pathway, the number of tissues in which it is significantly enriched in genes positively correlated with CAV1, and the number of tissues in which it is significantly correlated with genes inversely correlated with CAV1, are provided in parentheses. The upper row, depicted in shades of brown, represents the mean CAV1 level; the darker shades of purple indicate higher CAV1 levels. An asterisk designates whether that tissue expresses high or low CAV1 levels, in accordance with the clustering shown in Figure 1.

We next explored the landscape of upstream transcriptional regulators associated with CAV1 TSCVs employing FGSEA on ChIP-Seq datasets from the TFEA.ChIP [31] database, which integrates results from ReMap [32] and GeneHancer [33], among other sources. Unlike the GTEx transcriptomic data derived from human post-mortem samples, these ChIP-seq datasets are compiled from in vitro, cell line-based studies, providing complementary evidence about potential regulatory factors, rather than merely capturing steady-state transcript levels. It must be considered that several significant peaks identified in ChIP-seq datasets may not be functionally relevant in a given tissue context, and that direct binding to the CAV1 locus or the presence of specific motifs near it does not guarantee functional regulation at all the stages of cellular development. We, therefore, adopted a systems biology perspective that considers the co-regulation patterns of gene sets bound by transcriptional regulators (TRs). In other words, we focused on whether genes collectively targeted by a TR are also co-expressed with CAV1-regulated genes in a given tissue, providing a more general hypothesis-generating framework rather than definitive evidence of direct binding. This allowed us to pinpoint significant associations across various tissues. Our approach involved a meta-enrichment analysis to refine and identify pivotal transcriptional regulators, leading to the discovery of key clusters with distinct regulatory roles. The results obtained are shown in Figure 6.

Interestingly, chromatin modifiers emerged as components of CAV1-associated regulatory networks across tissues, with SMARCA2 showing a ubiquitous positive correlation with CAV1. In contrast, PRC2 members displayed tissue-specific correlations. The regulators involved in mechanobiology correlate positively with CAV1 in all the tissues (TEAD4), whereas others involved in immunity (RELA and STAT3), and metabolism (MYC, WDR5, NRF1, and GABPA) also show tissue-specific significant associations.

Clustering of the identified regulators reflected coherent functional grouping ranging from mitochondrial regulation and immune response to mechanoadaptation. Of note, brain tissues (known to have low CAV1 expression levels) exhibited unique negative correlations with PRC2 complex components, which are involved in brain tissue identity and blood–brain barrier homeostasis ([34,35]; see below).

Our upstream regulator profiling might support potential chromatin configuration shifts influencing CAV1 TSCVs, stressing the relationship between CAV1 regulation and downstream functions with cell state and identity. While these findings suggest potential upstream regulatory influences, they remain hypothesis-generating. Chromatin modifiers and other TRs often target hundreds or thousands of genes, and correlation alone cannot ultimately prove direct causality. As such, these observations must be experimentally validated to confirm functional relevance, guiding future mechanistic investigations into the regulation of CAV1 and its downstream effects.

#### 2.3.3. CAV1 Tissue Expression and Cell Type-Specific Regulatory Influence

**Figure 7:**
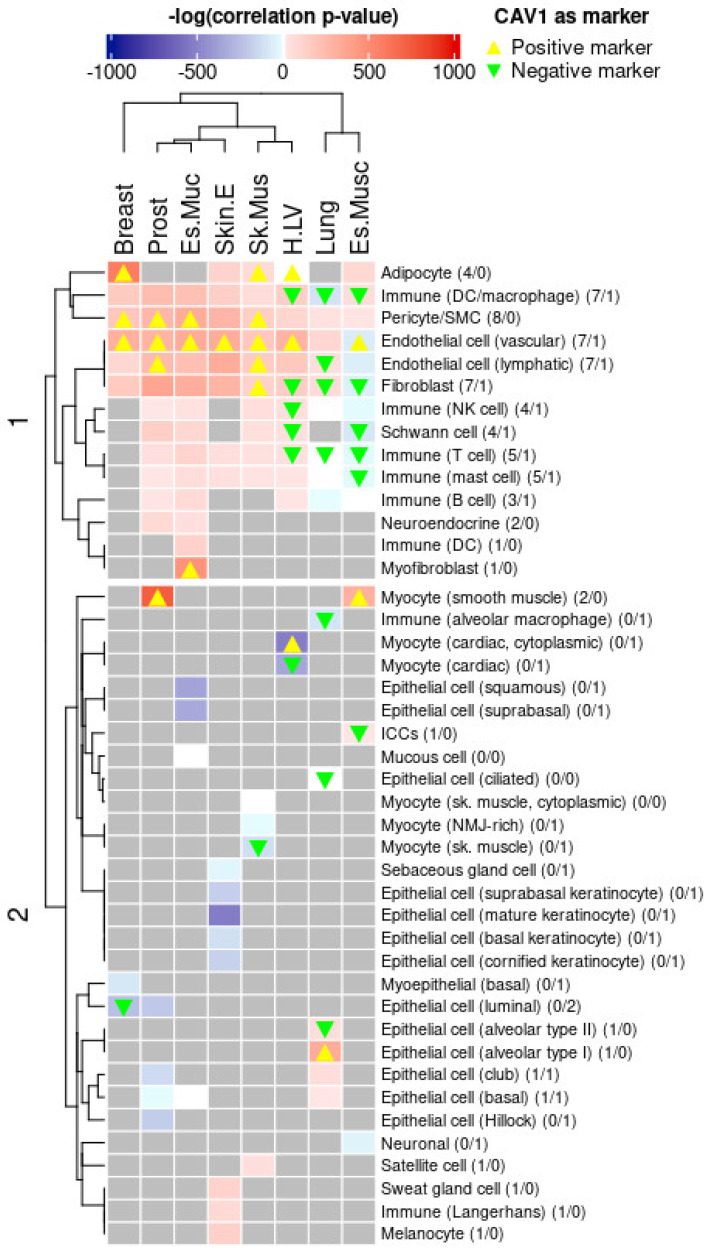
CAV1 TSCV enrichment results with cell type-specific markers obtained from single-nuclei GTEx reanalysis. Cell types whose markers are enriched in genes that directly correlate with CAV1 in that tissue are depicted in red. In blue, cell types whose markers are enriched in genes that inversely correlate with CAV1 in a given tissue. A yellow rectangle is added to cell type–tissue combinations where CAV1 is identified as a specific positive marker and a green rectangle is CAV1 is identified as a negative marker.

In order to further infer CAV1 tissue-level functions, we analyzed its association with different cell types, thereby revealing the dominant cell type regulatory programs linked to CAV1 in each tissue. To achieve this, we conducted an FGSEA of cell type markers from the GTEx snRNA-seq data, assessing CAV1 TSCVs against these markers. By examining the enrichment of cell type-specific marker sets among the genes correlated with CAV1, we aimed to identify which cellular populations might indirectly shape or reflect CAV1-related expression patterns. This approach helped control the impact of each cell type on the TSCVs. The results are shown in the heatmap of Figure 7. The relative abundance of cell types typically expressing CAV1, such as endothelial cells, adipocytes, and smooth muscle cells, showed a consistent positive correlation with CAV1 across most tissues. In contrast, the predicted abundance of cell types where CAV1 is usually negative or minimally present, like certain neurons or erythroblasts, displayed a more variable correlation with tissue CAV1 levels.

Notably, many immune cell markers positively correlated with CAV1-associated gene sets despite a consistent residual expression of CAV1 itself in these immune lineages. This unexpected finding suggests that CAV1 could serve as an indirect indicator of immune infiltration or immune-modulated tissue states. In other words, even though CAV1 is not expressed by these immune cells themselves, high CAV1 levels in bulk tissue samples may reflect or influence the presence and activity of immune cell populations. This relationship implies a potentially overlooked role for CAV1 in mediating crosstalk between different cell types and the microenvironment, including inflammatory processes and tissue remodeling events. By highlighting these associations, we underscore that CAV1 levels may provide insights into broader tissue dynamics, rather than functioning merely as a cell type-restricted marker. Our findings emphasize CAV1′s multifaceted role in tissue organization and remodeling upon different cues, including inflammation; they also highlight the need to consider cell type composition to fully understand CAV1′s diverse functions and regulatory mechanisms across different tissues. This perspective extends the interpretation of tissue-level CAV1 regulation beyond a unique cell compartment, and suggests that CAV1 expression patterns could help integrate complex tissue behaviors, such as immune infiltration, cellular adaptation, and the interplay of multiple signaling pathways. Additionally, these findings underscore the unique value of leveraging ex vivo human datasets, as our integrative analytic framework enables the detection and interpretation of complex, context-dependent regulatory interactions that are challenging to fully capture in simplified model systems.

## 3. Discussion

The pleiotropic, contextual, and cell type-specific nature of CAV1 function, tied to cell–cell and cell–environment communication, poses a complexity that is challenging to unravel. Here, we report our observations by leveraging the natural variability in gene expression exclusively across human tissues using extensive data from the GTEx project. This human-specific approach, avoiding cross-species comparisons, represents a critical contribution to achieving a systems-level, multilayered understanding of CAV1 function directly relevant to human physiology. This integrative computational approach is the closest method available for causal inference in complex human in vivo data, as in vitro models of tissue complexity rarely capture the intricate interactions we sought to elucidate. This approach bears limitations: the use of post-mortem samples introduces variables that might affect gene expression profiles, thus potentially skewing the interpretation of CAV1 physiological roles. Further, our modeling of cell type proportions, while methodologically robust, relies on markers with a limited degree of specificity. Nonetheless, beyond any potential influence from variability introduced by the different sampling/handling procedures across tissues, which we have also specifically assessed, our studies predict the involvement of chromatin organizers in the upstream regulation of CAV1, linking specific cell processes with CAV1 regulation in a tissue-specific fashion. Moreover, upon modeling the contribution of relative cell type abundance to the tissue-level regulation of CAV1, we propose that CAV1 is a relevant modulator and biomarker of immune cell infiltration across different tissues. We fully acknowledge that our purely computational and observational approach limits our ability to establish definitive causal relationships. However, within the constraints of statistical associations, our stepwise modeling strategy represents a robust method for causal inference, allowing us to infer potential causal relationships by comparing models with and without key mediating or confounding variables.

The intricate regulatory landscape of CAV1, as predicted by our study, demonstrates a lack of uniform transcriptional regulatory programs across tissues with different caveolae densities. This further supports the context-dependent biological role of CAV1 and its engagement across different adaptive responses, which cannot be categorized solely based on caveolae density.

A remarkable finding from our study is the predominant role of cell type proportion in dictating CAV1 transcription levels. This outcome urges a cautious approach when interpreting cellular-level regulatory conclusions from GTEx data. It highlights the substantial reciprocal impact of cell identity on CAV1 regulation, suggesting that external stimuli affecting cell type proportions in tissues may have a more significant influence on our findings than factors directly targeting CAV1 levels. However, the consistent correlation of CAV1 with key caveolae components across tissue-specific correlation vectors underlines the relevance of these components in the regulation of CAV1. This aspect emphasizes the need for a meticulous consideration of cell type composition in the functional and regulatory analysis of CAV1. A further specific insight from this approach is the prediction of CAV1 tissue-level expression as correlating and potentially driving immune cell infiltration and the dynamics of inflammatory processes. This is particularly relevant when considering the dynamics of inflammation upon tissue damage, and their coordination with those of the processes involved in tissue repair such as ECM deposition and remodeling, and cell phenotype transitions such as epithelial-to-mesenchymal transition (EMT). Indeed, the literature robustly links the CAV1 function to these events [36,37,38]. Additionally, these findings underscore the unique value of leveraging ex vivo human datasets, as our integrative analytic framework enables the detection and interpretation of complex, context-dependent regulatory interactions that are challenging to fully capture in simplified model systems.

Importantly, our analysis revealed that terminal phase context was among the chief determiners of CAV1 expression. This variable captures acute, tissue-specific conditions at the end of life that directly impact physiological states and CAV1 expression, leading to stronger associations compared to more gradual factors like age. Terminal phase context reflects biologically relevant end-of-life conditions, such as the absence of mechanical stimulation in ventilated individuals affecting muscle tissues or metabolic shifts in adipose tissues during end-of-life conditions. These findings suggest that terminal phase context influences tissue-specific physiological states, ultimately impacting CAV1 expression. In contrast, age exerts a more gradual, systemic influence, often mediated by tissue composition, which may explain its weaker independent associations. Regarding RIN, while it initially appeared to be significantly associated with CAV1 levels in multiple tissues, its significance was substantially reduced once we incorporated cell type proportions and additional phenotypic variables into the models, as shown in Table 1. In contrast, the variables related to the terminal phase context retained their influence even after these adjustments, suggesting that their effects are at least partly independent of tissue composition. Our interpretation is that the residual association of RIN observed in some tissues, even after accounting for cell type composition and other phenotypic factors, may reflect subtle technical or biological influences not fully captured by our current modeling approach. For example, variations in RNA quality among specific cell populations or differences in tissue handling could persist despite controlling for cell type proportions. Thus, while cell type composition explains much of the initial RIN-CAV1 correlation, a minor residual RIN effect could remain in certain cases, potentially reflecting tissue-specific factors. These observations emphasize the importance of considering both technical and biological confounders when interpreting gene expression data from post-mortem samples.

Moreover, our study underscores the importance of chromatin modifiers, such as SMARCA2, and tissue-specific regulators like PRC2 members, in the governance of CAV1 expression. The involvement of regulators integral to mechanobiology, immunity, and metabolism adds another dimension to the complex regulatory network surrounding CAV1. This aspect of our findings points to a fine-tuned, contextual interplay between various biological processes and CAV1 regulation, further complicating the narrative of CAV1′s role in cellular physiology.

In summary, our research not only contributes to a better understanding of CAV1 roles across diverse physiological contexts but also provides a framework for future investigations, exclusively sourced from human tissue data from the GTEx project. By focusing solely on human samples, avoiding inter-species extrapolation, we uncover regulatory networks with potential implications for pathologies involving inflammation and cell communication, offering novel opportunities for tailored precision medicine directly informed by human biology. These studies will be crucial for translating our computational findings into mechanistic insights and strategies with therapeutic intervention potential.

## 4. Materials and Methods

### 4.1. The Genotype-Tissue Expression (GTEx) Project Data

Bulk RNA-seq data from 17,382 human samples, spanning 52 different tissues and two in vitro cell cultures (including human fibroblasts, “Fibr”, and Epstein–Barr transformed human lymphocytes, “Lymph.T”), were used. Data were retrieved from the latest GTEx V8 release [27]. Gene-level Transcripts Per Million (TPM) data were downloaded from the GTEx Portal (https://www.gtexportal.org/home/datasets, accessed on 14 October 2022). The characteristics and abbreviations used across manuscript panels of these samples are summarized in Supplementary Table S1. Tissues with less than 25 samples were discarded due to low sampling size. In order to maintain consistency with gene symbol-annotated data in the MSigDB R package (version 1.12.0) [39], the GTEx GENCODE 26 gene symbol annotation was updated using the R packages ‘annotate’ (version 1.74.0), ‘org.Hs.eg.db’ (version 3.15.0), and ‘HGNChelper’ (version 0.8.1).

### 4.2. Defining Cell Type Markers Using GTEx snRNA-Seq Data

We used the 25 publicly accessible human single-nuclei RNA-seq (snRNA-seq) data samples from GTEx Analysis V9 (dbGaP Accession phs000424.v9) extracted from 16 individuals across 8 tissues. We downloaded the data object, “GTEx_8_tissues_snRNAseq_atlas_071421.public_obs.h5ad”, which contains cell line identities and cell type annotations as per the original publication [40]. This object was then transformed into a Seurat R object for further processing using the Seurat R package (version 4.0.1).

The dataset included a median of 30,969 cells per tissue and 44 different cell types, with a median of 1154 cells per cell type. High interindividual variability was observed in the cell count of each cell type per tissue, leading us to pool all the individuals to increase the cell count for each cell type within a given tissue. We performed eight separate analyses to identify cell type-specific markers for the 44 cell types mentioned in the original publication. For that purpose, we used the FindMarkers function of the Seurat package, applying a Wilcoxon test to identify markers of each cell type. A Bonferroni-adjusted *p*-value of 0.05 was selected as the cut-off. Only positive markers were selected for further processing as they are less susceptible to the low sensitivity of snRNA-seq data.

For cell types found in several tissues, we established a consistent definition of markers. A gene was considered a marker if it was identified as such in half or more of the tissues where the cell type was detected. This consistent marker definition will be used for the enrichment analysis of CAV1 TSCVs. However, it is important to remember that marker definitions are highly tissue- and sample-specific and influenced by cell type population size and the identity of other cell types in the sample.

### 4.3. Estimation of Relative Cell Type Proportion for Each Sample

Cell types identified in the snRNA-seq of each tissue were considered for relative cell type proportion estimation. The markers defined previously were used to estimate the cell type proportion in a tissue sample. We assumed that most identified markers were cell type-specific, and their relative quantity would directly correspond to the proportion of that cell type within the sample. However, a marker might also be expressed by other cell types at lower levels. To account for this, we calculated the 5% trimmed mean of genes identified as specific markers for a given cell type and examined its bivariate correlation with all the genes in the set. Genes with more than a 0.33 correlation with the trimmed mean were considered informative markers for estimating the cell type proportion.

For those tissues without available snRNA-seq data from GTEx, we considered a subset of the 44 identified cell types, excluding those not found in the tissue under consideration. Although we acknowledge the limitations of this approach, we followed it for consistency and reliance solely on GTEx data. We used a consistent set of markers for each cell type in this approach. The steps followed for cell type proportion estimation remained the same as those used for the eight tissues with available GTEx snRNA-Seq data.

### 4.4. Linear Modeling of CAV1 Expression: Assessing the Impact of Phenotypic Data and RNA Quality Metrics

Our objective was to delineate the primary sources of variability in CAV1 expression levels across tissues. For that, we fitted linear models to predict the expression of CAV1 through the different tissues and used three different sets of covariates as predictors. The covariates indicate different characteristics of the samples such as quality metrics, type of death, and others.

The multiple linear regression models are, hence, described by the following equation:

$$\vec{y}=X\;\vec{\beta }+\vec{\varepsilon }$$

Here, $\vec{y}$ is a column vector of the log2-transformed CAV1 expression values (log2[TPM]), X is the matrix of covariates of the GTEx variables, $\vec{\beta }$ represents the regression coefficients, and $\vec{\varepsilon }$ is a column vector of the error terms, assumed to be normally distributed, independent, and homoscedastic. The models were fitted using the lm function from the R stats package (version 4.2.1). A linear model was fitted to the samples for each tissue.

#### 4.4.1. Model Set 1: Using Publicly Available GTEx Sample Information

The initial model was fitted using only publicly accessible information about the samples. These variables are:

1. Essential RNA sample quality metrics:
  RIN (variable SMRIN): The RNA integrity number, a basic measure of the quality of isolated RNA.
  Autolysis Score (variable SMATSSCR): Estimation of the destruction of organism cells or tissues by the organisms’ own enzymes or processes. Determined by pathologists based on the visual inspection of histological images.
  Total Ischemic time for a sample (variable SMTSISCH): Interval between actual death, presumed death, or cross-clamp application and final tissue stabilization.
2. Phenotypic traits of the individual:
  Age (variable AGE_GROUP): Elapsed time since birth in decades: 20–29, 30–39, 40–49, 50–59, 60–69, and 70–79.
  Gender (variable SEX): Biological gender.
  Terminal Phase Context (variable DTHHRDY): DTHHRDY is a classification of death adapted from the 4-point Hardy Scale, indicating the circumstances and immediacy of the subject’s death, from sudden and violent deaths to slow deaths after a long illness. For more details, see Supplementary Table S2 in the Supplementary Materials.

The correlation between some variables, such as the terminal phase context (DTHHRDY) and the ischemic time (SMTSISCH), is significant within certain tissues, yet this does not pose any issues in model fitting, providing meaningful and robust results.

#### 4.4.2. Model Set 2: Adding Sample Relative Cell Type Proportions

To determine whether cell type proportions significantly impact sample CAV1 levels, we incorporated these proportions as predictors into this model set. We included the estimated relative cell type proportions for cell types that could potentially exist in the examined tissues. A comprehensive list of the cell types considered, as well as the number of tissues where they were utilized as predictors, is provided in Supplementary Table S2 in the Supplementary Materials.

The high correlation among cell types within the same tissues led to nonsensical model output. To manage multicollinearity, we implemented a feature selection based on an initial elastic net model. The elastic net was chosen as the initial feature selection mechanism due to its key properties as a regularization tool capable of dealing with large numbers of features exhibiting multicollinearity. This enabled us to select a subset of less-correlated, significant features for multiple linear regression modeling. Fitting an elastic net model involves finding the optimal β coefficients that minimize the following expression:

$$\hat{\beta }=\underset{\beta }{arg\;min}\;(\|y-X\beta {\|}^{2}+\lambda \;[(1-\alpha )\;\|\beta {\|}^{2}/2+\alpha \;\|\beta {\|}_{1}])$$

Here, the ‖ ‖_1_ operator denotes the L1 norm, the ‖ ‖^2^ operator denotes the Euclidean L2 norm, y is an n by 1 column vector of the log2(TPM) expression values of CAV1 for the n samples of the tissue being modeled, X is an n by (p + 1) matrix of the independent variable observations associated with the GTEx variables and cell type relative proportions, β is a (p + 1) by 1 column vector of the regression coefficients, λ is the parameter controlling the overall strength of the regression penalty, and α is the parameter adjusting the balance between the L1 and L2 penalties, ranging from lasso regression (α = 1) to ridge regression (α = 0).

This model was fitted using the caret (version 6.0-93) and glmnet (version 4.1-4) R packages. Hyperparameter optimization was carried out via 10-fold cross-validation, repeating each model 5 times, using the trainControl function from the caret R package. Optimization of the α and λ parameters was conducted using the tuneGrid parameter and glmnet method from the train function, both of which are components of the caret R package. We enforced elastic net behavior by optimizing α values within the [0.4, 0.6] range and ensured a positive penalty in all the models by optimizing λ within the [0.05, 0.2] range.

#### 4.4.3. Model Set 3: Using All GTEx Covariates

A new model was estimated using all the GTEx covariates (refer to Supplementary Table S2 in the Supplementary Materials for details). Predictors with more than 5% missing values were excluded from the analysis. For the remaining variables with missing values, we used the miceRanger function from the R package of the same name for imputation. This function employs MICE (Multiple Imputation by Chained Equations) for missing data imputation [41]. We noted considerable consistency across different imputation rounds. For the sake of reproducibility and computational reasons, only one dataset was imputed with a fixed seed and a maximum of 10 iterations. All the predictive variables that were not estimated as 0 in the elastic net model were included as predictors in the final linear regression model.

### 4.5. Estimation of CAV1 Tissue-Specific Correlation Vectors (TSCVs)

We used the GTEx project human data outlined in Supplementary Table S1 to construct a Tissue-Specific CAV1 Correlation Vector (TSCV) for each tissue analyzed. The steps taken are as follows:

(1) Initial data filtration retained only those transcriptional elements that exhibited at least 4 TPMs across over 5% of the tissue-specific samples.
(2) Normalization using the Trimmed Mean of M-values (TMM) algorithm [42]: This process was executed using the edgeR (version 3.38.4) R package [43], and the data were log-transformed.
(3) CAV1 TSCVs were estimated by determining the pairwise Pearson correlation coefficient between each transcriptional element (g) expressed in tissue (t) and the CAV1 levels in the same tissue. The formula used is as follows:
  $$r_{t,g}=\frac{\sum_{i=1}^{n}{(x}_{t,g,i}-{\overset{-}{x}}_{t,g}){(x}_{t,CAV1,i}-{\overset{-}{x}}_{t,CAV1})}{\sqrt{\sum_{i=1}^{n}{(x}_{t,g,i}-{\overset{-}{x}}_{t,g})}\sqrt{\sum_{i=1}^{n}\left(x_{t,CAV1,i}-{\bar{x}}_{t,CAV1}\right)}}$$
  where *r_t,g_* denotes the CAV1 TSCV for tissue t and gene g, $x_{t,g,i}$ represents the log2(TPM) expression of gene g in sample i of tissue t, $x_{t,CAV1,i}$ indicates the log2(TPM) expression of CAV1 sample i of tissue t, ${\overset{-}{x}}_{t,g}$ is the mean log2(TPM) expression of gene g in tissue t, and ${\overset{-}{x}}_{t,CAV1}$ is the mean log2(TPM) expression of CAV1 in tissue t.
(4) All the TSCVs were subsequently integrated into a unified matrix; R^T^:= (*r_g,t_*), a transpose of the aforementioned matrix and R:= (r_t,g_), consisting of 30,385 transcriptional elements expressed in at least one of the 54 tissues evaluated in GTEx. Each column of this matrix corresponds to the CAV1 TSCV from a specific tissue. Genes not expressed in a given tissue were assigned an NA value in this matrix. For gene-level PCA computation, a value of 0 must be assigned to those NA values to allow matrix inversion. It is important to note that the lack of evidence for a correlation due to the lack of expression of a gene does not imply a correlation of 0. Consequently, we employed alternative methods such as Multidimensional Scaling (MDS, see below) to ensure that significant biases were not introduced due to missing data.
(5) Tissues with less than 25 samples were eliminated from the analysis due to insufficient sample size. These included kidney medulla (*n* = 4), fallopian tube (*n* = 9), ectocervix (*n* = 9), endocervix (*n* = 10), and bladder (*n* = 21).

#### Multidimensional Scaling Analysis (MDS)

We applied Multidimensional Scaling (MDS) to reduce the dimensionality of the Tissue-Specific CAV1 Correlation Vectors (TSCVs) and to visualize relationships among tissues in a two-dimensional space. MDS is a statistical technique that starts from a distance or dissimilarity matrix—here, based on the pairwise correlation patterns between tissues—and finds a low-dimensional representation that preserves the relative distances as faithfully as possible.

In this context, each tissue was represented as a point in a multidimensional space defined by the correlations between CAV1 and all the expressed genes. MDS computed new orthogonal axes (“dimensions”) that captured the main sources of variation in these correlation profiles. Dimension 1 and Dimension 2 are the principal axes of variation chosen to maximize the amount of variance explained in the two-dimensional plot. It is important to note that these dimensions do not correspond to any single biological variable or factor. Instead, they are abstract constructs that summarize complex patterns of similarity and difference. Tissues positioned close to each other along these dimensions exhibit more similar CAV1 correlation patterns, while tissues that are far apart are more distinct in their CAV1-related transcriptional landscapes.

By projecting the data onto these two axes, MDS facilitated the interpretation of high-dimensional correlation structures and aided in identifying clusters of tissues with shared regulatory characteristics.

### 4.6. Functional and Upstream Regulator Enrichment of CAV1 TSCVs

Functional Enrichment Analysis: We utilized the Fast Gene Set Enrichment Analysis (FGSEA) method included in the fgsea R package (version 1.20.0) [44]. This efficient approach, an extension of the traditional GSEA technique, allowed us to calculate and normalize enrichment scores (ES) for each gene set based on a pre-ranked list of genes [45]. For KEGG gene annotations, we used the Molecular Signatures Database v7.5.1 [39].

Transcriptional Regulator Analysis: The Fast Gene Set Enrichment Analysis (FGSEA) method implemented in the fgsea R package [44] was also used. We employed the TFEA.ChIP database [31] for the association between targets and transcriptional regulators based on ChIP-seq data information. The TFEA.ChIP database, sourced from the ReMap 2022 database [32] and GeneHancer [33], covers numerous human ChIP-seq datasets.

Meta-Enrichment of Upstream Transcriptional Regulators: A secondary enrichment using FGSEA summarized results from the TFEA.ChIP matrix, producing a Gene Set Rank Statistic (GSRS) for each transcriptional regulator. This step involved synthesizing information from redundant ChIP-seq datasets corresponding to numerous unique transcriptional regulators.

## Figures and Tables

**Table 1:** Number of tissues in which each publicly available variable showed a statistically significant association (*p* < 0.05) with CAV1 expression under three different modeling scenarios. Death-related variables (in blue) include terminal phase context (the only death-related variable included in Model Sets 1 and 2 and terminal phase context, Death Manner, Death Place, Cohort (organ donor, post-mortem donor, or surgical donor), and Death Connected to a Ventilator from Model Set 3. Model Set 1 used only the listed technical and phenotypic variables (including RIN (in red), autolysis, ischemic time, age, sex, and terminal phase context). Model Set 2 incorporated cell type proportions in addition to the Model Set 1 variables, allowing us to assess how controlling for tissue composition influenced the significance of each variable. Model Set 3 further expanded the analysis by including additional phenotypic variables (described in Supplementary Table S1) while maintaining the cell type predictors identified in Model Set 2. This stepwise approach helped distinguish variables whose apparent influence on CAV1 expression was driven by underlying tissue composition (e.g., RIN) from those that remained significant even after accounting for cellular heterogeneity and more complex phenotypic information.

	Model Set 1	Model Set 2	Model Set 3
Death-related variables	17	15	16
RIN	15	4	4
Gender	5	2	1
Age	5	2	1
Ischemic time	3	0	1
Autolysis	2	0	0
PAXgene fixation time	0	0	0

## Data Availability

The data used for the analyses described in this manuscript were obtained from: the GTEx Portal on 14 October 2022 and dbGaP accession number phs000424.v9.pht002742.v9 on 14 October 2022.

## Supplementary Materials

The following supporting information can be downloaded at https://www.mdpi.com/article/10.3390/ijms26083789/s1.

## References

[1] Parton R.G., del Pozo M.A. (2013). Caveolae as plasma membrane sensors, protectors and organizers. Nat. Rev. Mol. Cell Biol..

[2] Palade G.E. (1953). Fine structure of blood capillaries. J. Appl. Phys..

[3] Sotodosos-Alonso L., Pulgarín-Alfaro M., del Pozo M.A. (2023). Caveolae Mechanotransduction at the Interface between Cytoskeleton and Extracellular Matrix. Cells.

[4] Glenney J.R. (1989). Tyrosine phosphorylation of a 22-kDa protein is correlated with transformation by Rous sarcoma virus. J. Biol. Chem..

[5] Glenney J.R., Zokas L. (1989). Novel tyrosine kinase substrates from Rous sarcoma virus-transformed cells are present in the membrane skeleton. J. Cell Biol..

[6] Kurzchalia T.V., Dupree P., Parton R.G., Kellner R., Virta H., Lehnert M., Simons K. (1992). VIP21, a 21-kD membrane protein is an integral component of trans-Golgi-network-derived transport vesicles. J. Cell Biol..

[7] Rothberg K.G., Heuser J.E., Donzell W.C., Ying Y.S., Glenney J.R., Anderson R.G.W. (1992). Caveolin, a protein component of caveolae membrane coats. Cell.

[8] Glenney J.R., Soppet D. (1992). Sequence and expression of caveolin, a protein component of caveolae plasma membrane domains phosphorylated on tyrosine in Rous sarcoma virus-transformed fibroblasts. Proc. Natl. Acad. Sci. USA.

[9] Drab M., Verkade P., Elger M., Kasper M., Lohn M., Lauterbach B., Menne J., Lindschau C., Mende F., Luft F.C. (2001). Loss of caveolae, vascular dysfunction, and pulmonary defects in caveolin-1 gene-disrupted mice. Science.

[10] Razani B., Combs T.P., Wang X.B., Frank P.G., Park D.S., Russell R.G., Li M., Tang B., Jelicks L.A., Scherer P.E. (2002). Caveolin-1-deficient mice are lean, resistant to diet-induced obesity, and show hypertriglyceridemia with adipocyte abnormalities. J. Biol. Chem..

[11] Zhao Y.-Y., Liu Y., Stan R.-V., Fan L., Gu Y., Dalton N., Chu P.-H., Peterson K., Ross J., Chien K.R. (2002). Defects in caveolin-1 cause dilated cardiomyopathy and pulmonary hypertension in knockout mice. Proc. Natl. Acad. Sci. USA.

[12] Fra A.M., Williamson E., Simons K., Parton R.G. (1995). De novo formation of caveolae in lymphocytes by expression of VIP21-caveolin. Proc. Natl. Acad. Sci. USA.

[13] Engelman J.A., Zhang X.L., Galbiati F., Lisanti M.P. (1998). Chromosomal localization, genomic organization, and developmental expression of the murine caveolin gene family (Cav-1, -2, and -3): Cav-1 and Cav-2 genes map to a known tumor suppressor locus (6-A2/7q31). FEBS Lett..

[14] Hagiwara Y., Sasaoka T., Araishi K., Imamura M., Yorifuji H., Nonaka I., Ozawa E., Kikuchi T. (2000). Caveolin-3 deficiency causes muscle degeneration in mice. Hum. Mol. Genet..

[15] Galbiati F., Engelman J.A., Volonte D., Zhang X.L., Minetti C., Li M., Hou H., Kneitz B., Edelmann W., Lisanti M.P. (2001). Caveolin-3 Null Mice Show a Loss of Caveolae, Changes in the Microdomain Distribution of the Dystrophin-Glycoprotein Complex, and T-tubule Abnormalities. J. Biol. Chem..

[16] Hill M.M., Bastiani M., Luetterforst R., Kirkham M., Kirkham A., Nixon S.J., Walser P., Abankwa D., Oorschot V.M., Martin S. (2008). PTRF-Cavin, a Conserved Cytoplasmic Protein Required for Caveola Formation and Function. Cell.

[17] Liu L., Pilch P.F. (2008). A critical role of cavin (polymerase I and transcript release factor) in caveolae formation and organization. J. Biol. Chem..

[18] Goetz J.G., Lajoie P., Wiseman S.M., Nabi I.R. (2008). Caveolin-1 in tumor progression: The good, the bad and the ugly. Cancer Metastasis Rev..

[19] Pol A., Morales-Paytuví F., Bosch M., Parton R.G. (2020). Non-caveolar caveolins—Duties outside the caves. J. Cell Sci..

[20] Mehta N., Zhang D., Li R., Wang T., Gava A., Parthasarathy P., Gao B., Krepinsky J.C. (2019). Caveolin-1 regulation of Sp1 controls production of the antifibrotic protein follistatin in kidney mesangial cells. Cell Commun. Signal..

[21] Zhang T., Hu Y., Wang T., Cai P. (2017). Dihydroartemisinin inhibits the viability of cervical cancer cells by upregulating caveolin 1 and mitochondrial carrier homolog 2: Involvement of p53 activation and NAD(P)H:quinone oxidoreductase 1 downregulation. Int. J. Mol. Med..

[22] Moreno-Vicente R., Pavón D.M., Martín-Padura I., Català-Montoro M., Díez-Sánchez A., Quílez-Álvarez A., López J.A., Sánchez-Álvarez M., Vázquez J., Strippoli R. (2018). Caveolin-1 Modulates Mechanotransduction Responses to Substrate Stiffness Through Actin-Dependent Control of YAP. Cell Rep..

[23] Samarakoon R., Chitnis S.S., Higgins S.P., Higgins C.E., Krepinsky J.C., Higgins P.J. (2011). Redox-Induced Src Kinase and Caveolin-1 Signaling in TGF-β1-Initiated SMAD2/3 Activation and PAI-1 Expression. PLoS ONE.

[24] Jiao L., Wang S., Zheng Y., Wang N., Yang B., Wang D., Yang D., Mei W., Zhao Z., Wang Z. (2019). Betulinic acid suppresses breast cancer aerobic glycolysis via caveolin-1/NF-κB/c-Myc pathway. Biochem. Pharmacol..

[25] Son Y.H., Lee S.-J., Lee K.-B., Lee J.-H., Jeong E.M., Chung S.G., Park S.-C., Kim I.-G. (2015). Dexamethasone downregulates caveolin-1 causing muscle atrophy via inhibited insulin signaling. J. Endocrinol..

[26] Dasari A., Bartholomew J.N., Volonte D., Galbiati F. (2006). Oxidative Stress Induces Premature Senescence by Stimulating Caveolin-1 Gene Transcription through p38 Mitogen-Activated Protein Kinase/Sp1–Mediated Activation of Two GC-Rich Promoter Elements. Cancer Res..

[27] Lonsdale J., Thomas J., Salvatore M., Phillips R., Lo E., Shad S., Hasz R., Walters G., Garcia F., Young N. (2013). The Genotype-Tissue Expression (GTEx) project. Nat. Genet..

[28] Domingues L., Hurbain I., Gilles-Marsens F., Sirés-Campos J., André N., Dewulf M., Romao M., Viaris de Lesegno C., Macé A.-S., Blouin C. (2020). Coupling of melanocyte signaling and mechanics by caveolae is required for human skin pigmentation. Nat. Commun..

[29] Catalán V., Gómez-Ambrosi J., Rodríguez A., Silva C., Rotellar F., Gil M.J., Cienfuegos J.A., Salvador J., Frühbeck G. (2008). Expression of caveolin-1 in human adipose tissue is upregulated in obesity and obesity-associated type 2 diabetes mellitus and related to inflammation. Clin. Endocrinol..

[30] Al Madhoun A., Kochumon S., Haddad D., Thomas R., Nizam R., Miranda L., Sindhu S., Bitar M.S., Ahmad R., Al-Mulla F. (2023). Adipose Tissue Caveolin-1 Upregulation in Obesity Involves TNF-α/NF-κB Mediated Signaling. Cells.

[31] Puente-Santamaria L., Wasserman W.W., Del Peso L. (2019). TFEA.ChIP: A tool kit for transcription factor binding site enrichment analysis capitalizing on ChIP-seq datasets. Bioinformatics.

[32] Hammal F., De Langen P., Bergon A., Lopez F., Ballester B. (2022). ReMap 2022: A database of Human, Mouse, Drosophila and Arabidopsis regulatory regions from an integrative analysis of DNA-binding sequencing experiments. Nucleic Acids Res..

[33] Fishilevich S., Nudel R., Rappaport N., Hadar R., Plaschkes I., Iny Stein T., Rosen N., Kohn A., Twik M., Safran M. (2017). GeneHancer: Genome-wide integration of enhancers and target genes in GeneCards. Database.

[34] Sun Z.-W., Wang X., Zhao Y., Sun Z.-X., Wu Y.-H., Hu H., Zhang L., Wang S.-D., Li F., Wei A.-J. (2024). Blood-brain barrier dysfunction mediated by the EZH2-Claudin-5 axis drives stress-induced TNF-α infiltration and depression-like behaviors. Brain Behav. Immun..

[35] Ihezie S.A., Mathew I.E., McBride D.W., Dienel A., Blackburn S.L., Pandit P.K.T. (2021). Epigenetics in blood–brain barrier disruption. Fluids Barriers CNS.

[36] Goetz J.G., Minguet S., Navarro-Lérida I., Lazcano J.J., Samaniego R., Calvo E., Tello M., Osteso-Ibáñez T., Pellinen T., Echarri A. (2011). Biomechanical remodeling of the microenvironment by stromal caveolin-1 favors tumor invasion and metastasis. Cell.

[37] Strippoli R., Sandoval P., Moreno-Vicente R., Rossi L., Battistelli C., Terri M., Pascual-Antón L., Loureiro M., Matteini F., Calvo E. (2020). Caveolin1 and YAP drive mechanically induced mesothelial to mesenchymal transition and fibrosis. Cell Death Dis..

[38] Cao J., Navis A., Cox B.D., Dickson A.L., Gemberling M., Karra R., Bagnat M., Poss K.D. (2016). Single epicardial cell transcriptome sequencing identifies Caveolin 1 as an essential factor in zebrafish heart regeneration. Development.

[39] Liberzon A., Subramanian A., Pinchback R., Thorvaldsdóttir H., Tamayo P., Mesirov J.P. (2011). Molecular signatures database (MSigDB) 3.0. Bioinformatics.

[40] Eraslan G., Drokhlyansky E., Anand S., Fiskin E., Subramanian A., Slyper M., Wang J., Van Wittenberghe N., Rouhana J.M., Waldman J. (2022). Single-nucleus cross-tissue molecular reference maps toward understanding disease gene function. Science.

[41] van Buuren S., Groothuis-Oudshoorn K. (2011). mice: Multivariate Imputation by Chained Equations in R. J. Stat. Softw..

[42] Robinson M.D., Oshlack A. (2010). A scaling normalization method for differential expression analysis of RNA-seq data. Genome Biol..

[43] Robinson M.D., McCarthy D.J., Smyth G.K. (2010). edgeR: A Bioconductor package for differential expression analysis of digital gene expression data. Bioinformatics.

[44] Korotkevich G., Sukhov V., Budin N., Shpak B., Artyomov M.N., Sergushichev A. (2016). Fast gene set enrichment analysis. Biorxiv.

[45] Subramanian A., Tamayo P., Mootha V.K., Mukherjee S., Ebert B.L., Gillette M.A., Paulovich A., Pomeroy S.L., Golub T.R., Lander E.S. (2005). Gene set enrichment analysis: A knowledge-based approach for interpreting genome-wide expression profiles. Proc. Natl. Acad. Sci. USA.

