# Supplementary material for: A Survey for Human Tissue-Level Determinants of CAV1 Regulation and Function
Source: Int J Mol Sci. 2025 Apr 17;26(8):3789. doi: 10.3390/ijms26083789 (PMC12027754; doi:10.3390/ijms26083789)
Supplement: Supplementary file 1 [file ijms-26-03789-s001.zip › ijms-3545080-supplementary.pdf]

Supplementary Materials

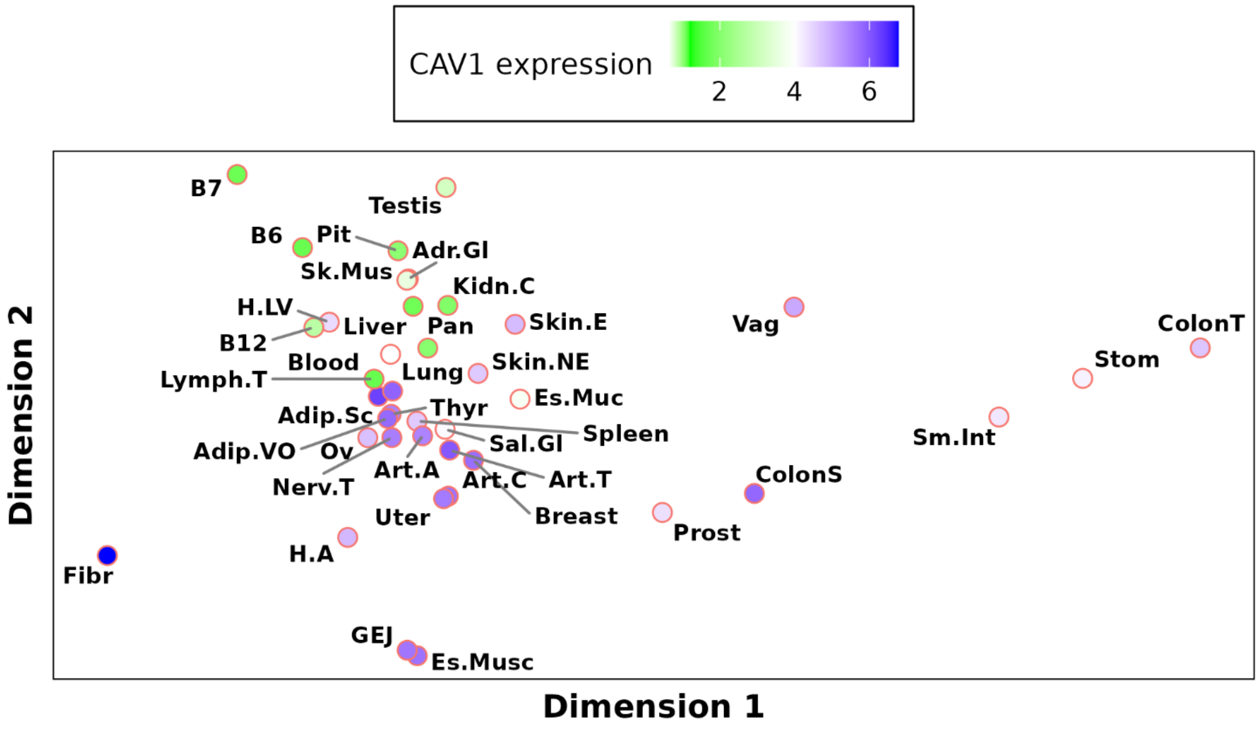

Supplementary Figure S1. MDS plot of CAV1 TSCVs upon brain anatomical region subsampling. No brain cluster can be recognized in this MDS plot.

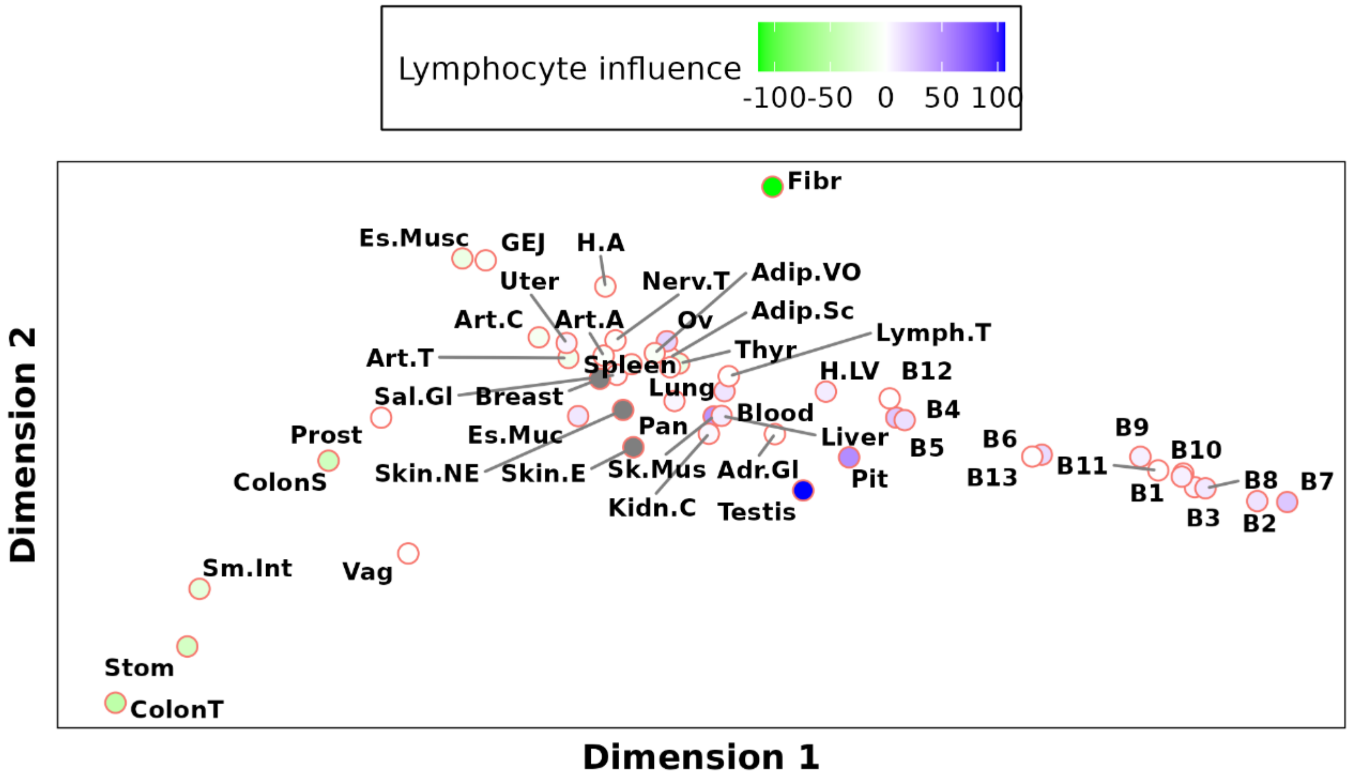

Supplementary Figure S2. Lymphocyte Influence on CAV1 Prediction in MDS Plot. This visualization presents the correlation between CAV1 levels and NK cell type proportions in various tissues. Tissues where CAV1 levels are

positively correlated with NK cell content are shown in blue, whereas tissues with an inverse correlation are depicted in green. Tissues where NK cell content does not significantly influence CAV1 expression are represented in white. Regions devoid of NK cells, indicating no impact on CAV1 prediction, are marked in grey."

Supplementary Table S1 *(GTEx samples). Tissues in the GTEx project and the number of samples available for each tissue. In red, tissues that were removed from the analysis due to low sample size (less than 25 samples).*

Tissue	**Labels**	**Sample size**
Subcutaneous adipose tissue	Adip.Sc	663
Adipose tissue from visceral omentum	Adip.VO	541
Adrenal gland	Adr.Gl	258
Aorta artery	Art.A	432
Coronary artery	Art.C	240
Tibial artery	Art.T	663
Bladder	Bladder	21
Brain amygdala	B1	152
Brain anterior cingulate cortex (BA24)	B2	176
Brain caudate (basal ganglia)	B3	246
Brain cerebellar hemisphere	B4	215
Brain cerebellum	B5	241
Brain cortex	B6	255
Brain frontal cortex (BA9)	B7	209
Brain hippocampus	B8	197
Brain hypothalamus	B9	202
Brain nucleus accumbens (basal ganglia)	B10	246
Brain putamen (basal ganglia)	B11	205
Brain spinal cord (cervical, c-1)	B12	159
Brain substantia nigra	B13	139
Breast mammary tissue	Breast	459
Cultured fibroblasts	Fibr	504
EBV-transformed lymphocytes	Lymph.T	174
Ectocervix	Cerv1	9
Endocervix	Cerv2	10
Sigmoid colon	ColonS	373
Transverse colon	ColonT	406
**Tissue**	**Labels**	**Sample size**
Esophagus gastroesophageal junction	GEJ	375
Esophagus mucosa	Es.Muc	555
Esophagus muscularis	Es.Musc	515
Fallopian tube	FT	9

Heart atrial appendage	H.A	429
Heart left ventricle	H.LV	432
Kidney cortex	Kidn.C	85
Kidney medulla	Kidn.M	4
Liver	Liver	226
Lung	Lung	578
Minor salivary gland	Sal.Gl	162
Skeletal muscle	Sk.Mus	803
Tibial nerve	Nerv.T	619
Ovary	Ov	180
Pancreas	Pan	328
Pituitary	Pit	283
Prostate	Prost	245
Skin, not Sun exposed (suprapubic)	Skin.NE	604
Skin, Sun exposed (lower leg)	Skin.E	701
Small intestine terminal ileum	Sm.Int	187
Spleen	Spleen	241
Stomach	Stom	359
Testis	Testis	361
Thyroid	Thyr	653
Uterus	Uter	142
Vagina	Vag	156
Whole blood	Blood	755

Supplementary Table S2 *(GTEx variables). Variables utilized in GTEx CAV1 linear predictive models. This table provides descriptions of variables used in the linear predictive models for CAV1, the number of tissues where each model was applied, and the specific set of models in which each variable was considered. Variable exclusions may have been due to a lack of data for the given variable in certain tissues or an insufficient variability in values (at least 5% of the samples should have a value differing from the most common one).*

Variable Name	Description	Nº	Model Sets
AGE_GROUP	Elapsed time since birth in decades: 20-29, 30-39, 40-49, 50-59, 60-69, 70-79.	49	1, 2, 3
DTHHRDY	Death classification based on the GTEx-adapted 4-point Hardy Scale:<br>1) Violent, fast death Deaths due to accident, blunt force trauma or suicide, terminal phase estimated at < 10 min.<br>2) Fast death of natural causes Sudden unexpected deaths of people who had been reasonably healthy, after a terminal phase estimated at < 1 hr. (with sudden death from a myocardial infarction as a model cause of death for this category)<br>3) Intermediate death after a terminal phase of 1 to 24 hrs. (not classifiable as 2 or 4); patients who were ill, but death was unexpected<br>4) Slow death after a long illness, with a terminal phase longer than 1 day (commonly cancer or chronic pulmonary disease); deaths that are not unexpected<br>0) Ventilator Case All cases on a ventilator immediately before death.	48	1, 2, 3
SEX	The donor's identification of sex based upon self-report, family/next of kin, or medical record abstraction.	44	1, 2, 3
SMATSSCR	Autolysis Score: Estimation of the destruction of organism cells or tissues by the organisms' own enzymes or processes.	35	1, 2, 3

Variable Name	Description	№	Model Sets
SMRIN	RIN Number: The RNA Integrity Number, a basic measure of the quality of RNA isolated	49	1, 2, 3
SMTSISCH	Total Ischemic time for a sample: Interval between actual death, presumed death, or cross clamp application, final tissue stabilization	49	1, 2, 3
SMTSPAX	Total time a sample spent in the PAXgene™ fixative	35	1, 2, 3
Adipocyte	Estimated sample relative cell type proportion	28	2, 3
Endothelial cell (lymphatic)	Estimated sample relative cell type proportion	35	2, 3
Endothelial cell (vascular)	Estimated sample relative cell type proportion	49	2, 3
Epithelial cell (alveolar type I)	Estimated sample relative cell type proportion	3	2, 3
Epithelial cell (alveolar type II)	Estimated sample relative cell type proportion	3	2, 3
Epithelial cell (basal)	Estimated sample relative cell type proportion	19	2, 3
Epithelial cell (basal keratinocyte)	Estimated sample relative cell type proportion	8	2, 3
Epithelial cell (ciliated)	Estimated sample relative cell type proportion	7	2, 3
Epithelial cell (club)	Estimated sample relative cell type proportion	4	2, 3
Epithelial cell (cornified keratinocyte)	Estimated sample relative cell type proportion	8	2, 3
Epithelial cell (Hillock)	Estimated sample relative cell type proportion	3	2, 3
Epithelial cell (luminal)	Estimated sample relative cell type proportion	13	2, 3
Epithelial cell (mature keratinocyte)	Estimated sample relative cell type proportion	8	2, 3
Epithelial cell (squamous)	Estimated sample relative cell type proportion	13	2, 3
Epithelial cell (suprabasal)	Estimated sample relative cell type proportion	17	2, 3
Epithelial cell (suprabasal keratinocyte)	Estimated sample relative cell type proportion	8	2, 3
Fibroblast	Estimated sample relative cell type proportion	33	2, 3
ICCs	Estimated sample relative cell type proportion	8	2, 3
Immune (alveolar macrophage)	Estimated sample relative cell type proportion	3	2, 3
Immune (B cell)	Estimated sample relative cell type proportion	45	2, 3
Immune (DC)	Estimated sample relative cell type proportion	40	2, 3
Immune (DC/macrophage)	Estimated sample relative cell type proportion	49	2, 3
Immune (Langerhans)	Estimated sample relative cell type proportion	4	2, 3
Immune (mast cell)	Estimated sample relative cell type proportion	34	2, 3
Immune (NK cell)	Estimated sample relative cell type proportion	46	2, 3
Immune (T cell)	Estimated sample relative cell type proportion	48	2, 3
Melanocyte	Estimated sample relative cell type proportion	4	2, 3
Mucous cell	Estimated sample relative cell type proportion	12	2, 3
Myocyte (cardiac)	Estimated sample relative cell type proportion	4	2, 3

Variable Name	Description	№	Model Sets
Myocyte (cardiac, cytoplasmic)	Estimated sample relative cell type proportion	4	2, 3
Myocyte (NMJ rich)	Estimated sample relative cell type proportion	3	2, 3
Myocyte (sk. muscle)	Estimated sample relative cell type proportion	3	2, 3
Myocyte (sk. muscle, cytoplasmic)	Estimated sample relative cell type proportion	3	2, 3
Myocyte (smooth muscle)	Estimated sample relative cell type proportion	23	2, 3
Myoepithelial (basal)	Estimated sample relative cell type proportion	8	2, 3
Myofibroblast	Estimated sample relative cell type proportion	17	2, 3
Neuroendocrine	Estimated sample relative cell type proportion	36	2, 3
Neuronal	Estimated sample relative cell type proportion	32	2, 3
Pericyte/SMC	Estimated sample relative cell type proportion	49	2, 3
Satellite cell	Estimated sample relative cell type proportion	3	2, 3
Schwann cell	Estimated sample relative cell type proportion	40	2, 3
Sebaceous gland cell	Estimated sample relative cell type proportion	4	2, 3
Sweat gland cell	Estimated sample relative cell type proportion	4	2, 3
Unknown	Estimated sample relative cell type proportion	4	2, 3
AGE	Age: Elapsed time since birth in years.	49	3
BMI	BMI: General indicator of the body fat an individual is carrying based upon the ratio of weight to height. BMI = 703 * weight in pounds/height in inches squared.	49	3
COHORT	Cohort: The study subgroup that the participant belongs to. This indicates whether the participant was a post mortem donor, an organ donor, or a surgical donor.	35	3
DTHMNNR	Manner of Death: An adverse event resulting in death.	48	3
DTHPLCE	Place of Death: Location where death occurred.	46	3
DTHSEASON	Season of death: Season of death.	48	3
DTHVNT	Was donor on a ventilator immediately prior to Death?	47	3
HGHT	Height: The height of Donor in inches as reported by Donor, family/next of kin, or medical record abstraction.	49	3
MHABNWBC	Abnormal WBC: Did the Donor have a history of Abnormal White Blood Cell at time of death?	49	3
MHARTHTS	Arthritis: An inflammatory process affecting a joint. Causes include infection, autoimmune processes, degenerative processes, trauma. Symptoms may include swelling around the affected joint, pain.	49	3
MHASCITES	Ascites: Did the Donor have a history of Ascites at time of death?	10	3
MHASTHMA	Asthma: A chronic respiratory disease manifested as difficulty breathing due to the narrowing of bronchial passageways.	49	3
MHBCTINF	Bacterial Infections: An acute infectious disorder caused by gram positive or gram-negative bacteria. Representative examples include pneumococcal, streptococcal, salmonella, meningeal infections.	30	3
MHBLDDND	Has blood donation been denied in the past, specify below. Has the donor ever volunteered to donate blood, been denied?	36	3
MHBRNPH	pH of the Donor's brain, measured post mortem in the cerebellum.	14	3
MHBRNWGHT	Post mortem weight of the Donor's brain, in grams.	11	3
MHCANCERNM	Verification of whether the donor has ever been diagnosed with metastatic cancer as provided by the primary history source.	45	3
MHCLRD	Chronic Lower Respiratory Disease: Chronic Lower Respiratory Disease (CLRD) (chronic bronchitis, emphysema, asthma).	2	3
MHCOCAINE5	Cocaine use in the past 5 years	28	3

Variable Name	Description	№	Model Sets
MHCOPD	Chronic Respiratory Disease: A chronic, progressive lung disorder characterized by the loss of elasticity of the bronchial tree, the air sacs, destruction of the air sacs wall, thickening of the bronchial wall, mucous accumulation in the bronchial tree. The pathologic changes result in the disruption of the air flow in the bronchial airways. Symptoms include shortness of breath, wheezing, productive cough, chest tightness. The two main types of chronic obstructive pulmonary disease are chronic obstructive bronchitis, emphysema.	49	3
MHCVD	Cerebrovascular Disease: Disorder resulting from inadequate blood flow in the vessels that supply the brain. Representative examples include cerebrovascular ischemia, cerebral embolism, cerebral infarction.	49	3
MHDLYSIS	Dialysis treatment (long term greater than 1 month, 3 times per week): Did the donor have dialysis treatment for at least three times a week, for at least one month prior to death?	4	3
MHDPRSSN	Depression: A mood disorder having a clinical course involving one or more episodes of serious psychological depression that last two or more weeks each, do not have intervening episodes of mania or hypomania, are characterized by a loss of interest or pleasure in almost all activities, by some or all of disturbances of appetite, sleep, or psychomotor functioning, a decrease in energy, difficulties in thinking or making decisions, loss of self-esteem or feelings of guilt, suicidal thoughts or attempts.	48	3
MHDRNKSTS	Did the Donor drink?	40	3
MHHRTATT	Heart attack, acute myocardial infarction, acute coronary syndrome: Gross necrosis of the myocardium, as a result of interruption of the blood supply to the area, as in coronary thrombosis.	49	3
MHHRTDIS	Ischemic Heart Disease (coronary artery disease (CAD), coronary heart disease, ischemic cardiomyopathy): A disorder of cardiac function caused by insufficient blood flow to the muscle tissue of the heart. The decreased blood flow may be due to narrowing of the coronary arteries, to obstruction by a thrombus, or less commonly, to diffuse narrowing of arterioles, other small vessels within the heart. Severe interruption of the blood supply to the myocardial tissue may result in necrosis of cardiac muscle (myocardial infarction).	49	3
MHHRTDISB	Heart Disease (Idiopathic): Does the Donor have a history of Heart Disease (Idiopathic)?	1	3
MHHTN	Hypertension: Pathological increase in blood pressure; a repeatedly elevated blood pressure exceeding 140 over 90 mmHg.	49	3
MHLVRDIS	Liver Disease (liver abscess, failure, fatty liver syndrome, inherited liver insufficiency, acute/chronic hepatic insufficiency, necrobacillosis, rupture): A non-neoplastic or neoplastic disorder that affects the liver parenchyma, intrahepatic bile ducts. Representative examples of non-neoplastic disorders include hepatitis, cirrhosis, cholangitis, polycystic liver disease. Representative examples of neoplastic disorders include hepatocellular adenoma, hepatocellular carcinoma, intrahepatic cholangiocarcinoma, lymphoma, angiosarcoma.	39	3
MHNRTHEUR	Resided on a Northern European military base for 6 months from 1980-1990 or elsewhere in Europe from 1980-1996: Did the Donor reside on a Northern European military base for 6 months from 1980-1990 or elsewhere in Europe from 1980-1996.	1	3
MHOPNWND	Open Wounds: Did the Donor have a history of open wounds at time of death?	11	3

Variable Name	Description	№	Model Sets
MHORGNTP	Received a human and/or animal tissue and/or organ transplant or xenotransplant. Did the donor ever receive a human and/or animal tissue and/or organ transplant or xenotransplant?	24	3
MHPLLABS	Prescription pill use that are not prescribed to the donor: Does the Donor have a history of prescription pill use that are not prescribed to the donor?	11	3
MHPNMIAB	Pneumonia: Did the Donor have a history of Pneumonia at time of death?	43	3
MHPNMNIA	Pneumonia (acute respiratory infection affecting the lungs): An acute, acute, chronic, or chronic inflammation focally or diffusely affecting the lung parenchyma, due to infections (viruses, fungi, mycoplasma, or bacteria), treatment (e.g., radiation), or exposure (inhalation) to chemicals. Symptoms include cough, shortness of breath, fevers, chills, chest pain, headache, sweating, weakness.	45	3
MHRA	Rheumatoid Arthritis: Does the Donor have a history of Rheumatoid Arthritis?	1	3
MHRNLFLR	Renal Failure: Acute or chronic condition, characterized by the inability of the kidneys to adequately filter the blood substances, resulting in uremia, electrolyte imbalances. Acute renal failure is usually associated with oliguria or anuria, hyperkalemia, pulmonary edema. Chronic renal failure is irreversible, requires hemodialysis.	48	3
MHSMKSTS	Did the Donor smoke?	42	3
MHSZRSU	Unexplained seizures: Did the Donor have a history of unexplained seizures? Provided by Primary History Source.	3	3
MHT1D	Diabetes mellitus type 1 (IDDM, formerly juvenile diabetes): A metabolic disorder characterized by abnormally high blood sugar levels due to diminished production of insulin or insulin resistance/desensitization.	32	3
MHT2D	Diabetes mellitus type II (NIDDM, adult-onset diabetes): A type of diabetes mellitus that is characterized by insulin resistance or desensitization, increased blood glucose levels. This is a chronic disease that can develop gradually over the life of a patient, can be linked to both environmental factors, heredity.	49	3
MHTXCEXP	Exposure to toxic substances that may have led to chronic conditions: Does the donor have any known exposure to toxic substances that may have led to chronic conditions?	9	3
MHWTLSUA	Unexplained weight loss: Does the history, physical examination, medical records, or autopsy report show unexplained weight loss.	1	3
RACE	Race: Report of Donor's race as reported by Donor, family/next of kin, or medical record abstraction. These classification categories, taken from the NIH, refer to geographically based categories that humans share, either by common history, nationality, or geographic distribution.	49	3
TRCHSTIND	Chest Incision Time: Interval between actual death, presumed death, or cross clamp application, the time of chest incision (Unit is Minutes).	48	3
TRDNISCH	Donor Ischemic Time: Interval between actual death, presumed death, or cross clamp application, first tissue stabilization. A single donor-level ischemic time point at which the first tissue was collected for that donor.	49	3
TRISCHD	GTEX Procedure Start Time: Interval between actual death, presumed death, or cross clamp application, the start of the GTEx Procedure. Unit is Minutes.	48	3
TRTPTREF	Tissue Recovery Time Point Reference: Indicates whether the tissue ischemic time begins at the donor's actual death, presumed death, or when the cross clamp was applied in the case of surgical donors.	17	3

Variable Name	Description	№	Model Sets
WGHT	Weight: The weight of Donor in pounds as reported by Donor, family/next of kin, or medical record abstraction.	49	3
